# Nuclear power in the 21st century: Challenges and possibilities

**DOI:** 10.1007/s13280-015-0732-y

**Published:** 2015-12-14

**Authors:** Akos Horvath, Elisabeth Rachlew

**Affiliations:** MTA Centre for Energy Research, KFKI Campus, P.O.B. 49, Budapest 114, 1525 Hungary; Department of Physics, Royal Institute of Technology, KTH, 10691 Stockholm, Sweden

**Keywords:** Fission, Fusion, Fusion plasma physics, Nuclear power, Nuclear waste, Reactor physics

## Abstract

The current situation and possible future developments for nuclear power—including fission and fusion processes—is presented. The fission nuclear power continues to be an essential part of the low-carbon electricity generation in the world for decades to come. There are breakthrough possibilities in the development of new generation nuclear reactors where the life-time of the nuclear waste can be reduced to some hundreds of years instead of the present time-scales of hundred thousand of years. Research on the fourth generation reactors is needed for the realisation of this development. For the fast nuclear reactors, a substantial research and development effort is required in many fields—from material sciences to safety demonstration—to attain the envisaged goals. Fusion provides a long-term vision for an efficient energy production. The fusion option for a nuclear reactor for efficient production of electricity has been set out in a focussed European programme including the international project of ITER after which a fusion electricity DEMO reactor is envisaged.

## Introduction

All countries have a common interest in securing sustainable, low-cost energy supplies with minimal impact on the environment; therefore, many consider nuclear energy as part of their energy mix in fulfilling policy objectives. The discussion of the role of nuclear energy is especially topical for industrialised countries wishing to reduce carbon emissions below the current levels. The latest report from IPCC WGIII (2014) (see Box 1 for explanations of all acronyms in the article) says: “Nuclear energy is a mature low-GHG emission source of base load power, but its share of global electricity has been declining since 1993. Nuclear energy could make an increasing contribution to low-carbon energy supply, but a variety of barriers and risks exist*”.*

Demand for electricity is likely to increase significantly in the future, as current fossil fuel uses are being substituted by processes using electricity. For example, the transport sector is likely to rely increasingly on electricity, whether in the form of fully electric or hybrid vehicles, either using battery power or synthetic hydrocarbon fuels. Here, nuclear power can also contribute, via generation of either electricity or process heat for the production of hydrogen or other fuels.

In Europe, in particular, the public opinion about safety and regulations with nuclear power has introduced much critical discussions about the continuation of nuclear power, and Germany has introduced the “Energiewende” with the goal to close all their nuclear power by 2022. The contribution of nuclear power to the electricity production in the different countries in Europe differs widely with some countries having zero contribution (e.g. Italy, Lithuania) and some with the major part comprising nuclear power (e.g. France, Hungary, Belgium, Slovakia, Sweden).

## Current status

**Fig. 1:**
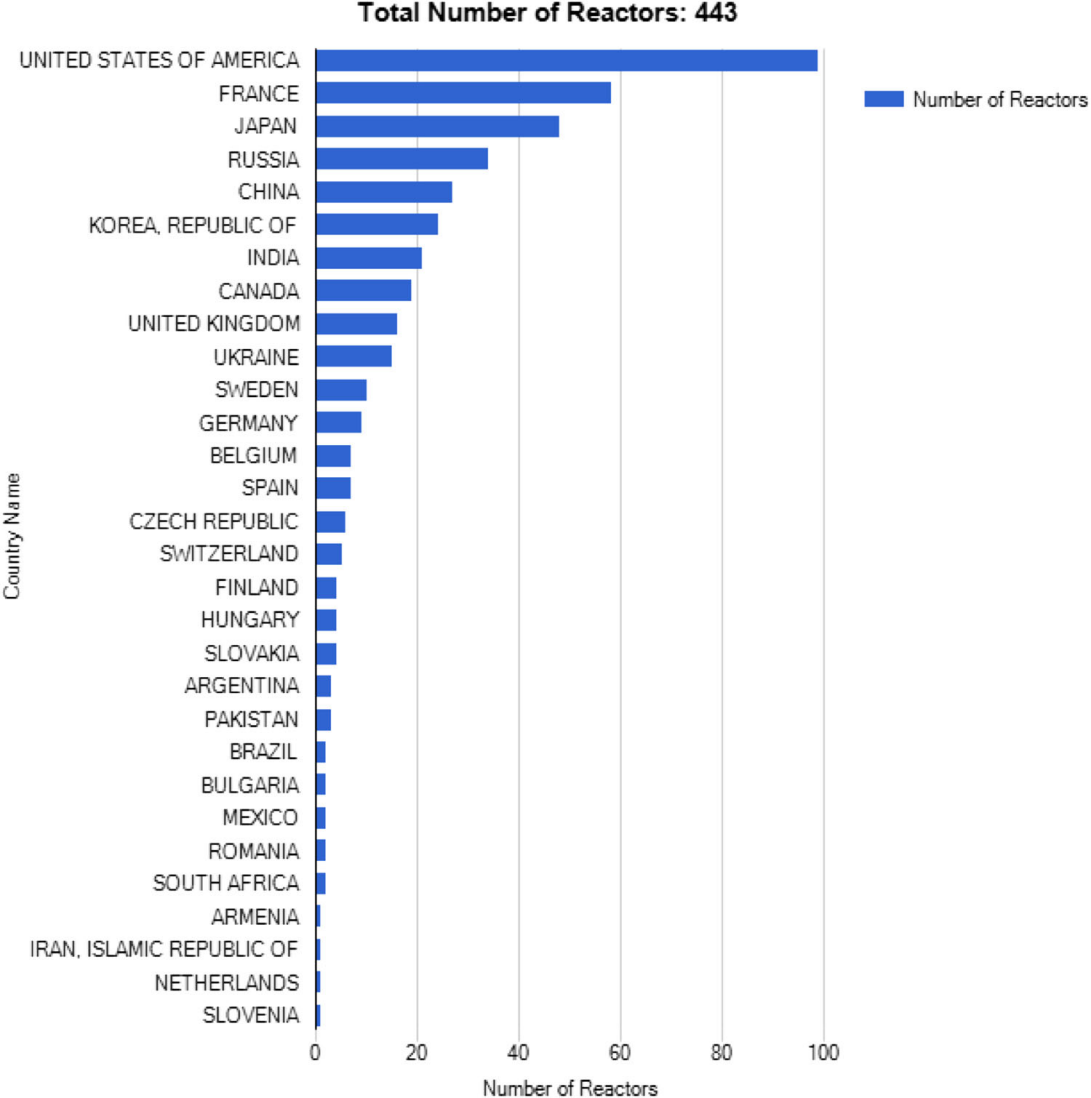
Total number of operating nuclear reactors worldwide. The total number of reactors also include six in Taiwan (source: IAEA 2015) (https://www.iaea.org/newscenter/focus/nuclear-power)

The use of nuclear energy for commercial electricity production began in the mid-1950s. In 2013, the world’s 392 GW of installed nuclear capacity accounted for 11 % of electricity generation produced by around 440 nuclear power plants situated in 30 countries (Fig. 1). This share has declined gradually since 1996, when it reached almost 18 %, as the rate of new nuclear additions (and generation) has been outpaced by the expansion of other technologies. After hydropower, nuclear is the world’s second-largest source of low-carbon electricity generation (IEA 2014,1).

The Country Nuclear Power Profiles (CNPP^2^) compiles background information on the status and development of nuclear power programmes in member states. The CNPP’s main objectives are to consolidate information about the nuclear power infrastructures in participating countries, and to present factors related to the effective planning, decision-making and implementation of nuclear power programmes that together lead to safe and economical operations of nuclear power plants.

Within the European Union, 27 % of electricity production (13 % of primary energy) is obtained from 132 nuclear power plants in January 2015 (Fig. 1). Across the world, 65 new reactors are under construction, mainly in Asia (China, South Korea, India), and also in Russia, Slovakia, France and Finland. Many other new reactors are in the planning stage, including for example, 12 in the UK.

**Fig. 2:**
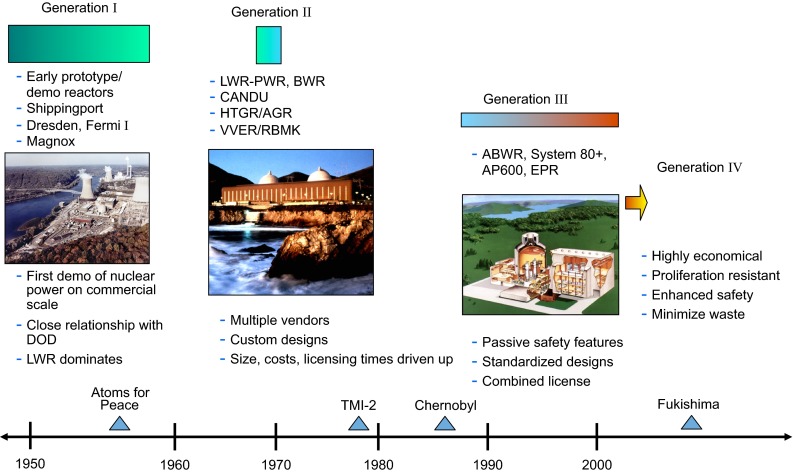
Nuclear reactor generations from the pioneering age to the next decade (reproduced with permission from Ricotti 2013)

Apart from one first Generation “Magnox” reactor still operating in the UK, the remainder of the operating fleet is of the second or third Generation type (Fig. 2). The predominant technology is the Light Water Reactor (LWR) developed originally in the United States by Westinghouse and then exploited massively by France and others in the 1970s as a response to the 1973 oil crisis. The UK followed a different path and pursued the Advanced Gas-cooled Reactor (AGR). Some countries (France, UK, Russia, Japan) built demonstration scale fast neutron reactors in the 1960s and 70s, but the only commercial reactor of this type currently operating is in Russia.

## Future evolution

### Introduction

The fourth Generation reactors, offering the potential of much higher energy recovery and reduced volumes of radioactive waste, are under study in the framework of the “Generation IV International Forum” (GIF)^3^ and the “International Project on Innovative Nuclear Reactors and Fuel Cycles” (INPRO). The European Commission in 2010 launched the European Sustainable Nuclear Industrial Initiative (ESNII), which will support three Generation IV fast reactor projects as part of the EU’s plan to promote low-carbon energy technologies. Other initiatives supporting biomass, wind, solar, electricity grids and carbon sequestration are in parallel. ESNII will take forward: the Astrid sodium-cooled fast reactor (SFR) proposed by France, the Allegro gas-cooled fast reactor (GFR) supported by central and eastern Europe and the MYRRHA lead- cooled fast reactor (LFR) technology pilot proposed by Belgium.

The generation of nuclear energy from uranium produces not only electricity but also spent fuel and high-level radioactive waste (HLW) as a by-product. For this HLW, a technical and socially acceptable solution is necessary. The time scale needed for the radiotoxicity of the spent fuel to drop to the level of natural uranium is very long (i.e. of the order of 200 000–300 000 years). The preferred solution for disposing of spent fuel or the HLW resulting from classical reprocessing is deep geological storage. Whilst there are no such geological repositories operating yet in the world, Sweden, Finland and France are on track to have such facilities ready by 2025 (Kautsky et al. 2013). In this context it should also be mentioned that it is only for a minor fraction of the HLW that recycling and transmutation is required since adequate separation techniques of the fuel can be recycled and again fed through the LWR system.

The “Strategic Energy Technology Plan” (SET-Plan) identifies fission energy as one of the contributors to the 2050 objectives of a low-carbon energy mix, relying on the Generation-3 reactors, closed fuel cycle and the start of implementation of Generation IV reactors making nuclear energy more sustainable. The EU Energy Roadmap 2050 provides decarbonisation scenarios with different assumptions from the nuclear perspective: two scenarios contemplate a nuclear phase-out by 2050, whilst three others consider that 15–20 % of electricity will be produced by nuclear energy. If by 2050 a generation capacity of 20 % nuclear electricity (140 GWe) is to be secured, 100–120 nuclear power units will have to be built between now and 2050, the precise number depending on the power rating (Garbil and Goethem 2013).

Despite the regional differences in the development plans, the main questions are of common interest to all countries, and require solutions in order to maintain nuclear power in the power mix of contributing to sustainable economic growth. The questions include (i) maintaining safe operation of the nuclear plants, (ii) securing the fuel supplies, (iii) a strategy for the management of radioactive waste and spent nuclear fuel.

Safety and non-proliferation risks are managed in accordance with the international rules issued both by IAEA and EURATOM in the EU. The nuclear countries have signed the corresponding agreements and the majority of them have created the necessary legal and regulatory structure (Nuclear Safety Authority). As regards radioactive wastes, particularly high-level wastes (HLW) and spent fuel (SF) most of the countries have long-term policies. The establishment of new nuclear units and the associated nuclear technology developments offer new perspectives, which may need reconsideration of fuel cycle policies and more active regional and global co-operation.

### Open and closed fuel cycle

In the frame of the open fuel cycle, the spent fuel will be taken to final disposal without recycling. Deep geological repositories are the only available option for isolating the highly radioactive materials for a very long time from the biosphere. Long-term (80–100 years) near soil intermediate storages are realised in e.g. France and the Netherlands which will allow for permanent access and inspection. The main advantage of the open fuel cycle is its simplicity. The spent fuel assemblies are first stored in interim storage for several years or decades, then they will be placed in special containers and moved into deep underground storage facilities. The technology for producing such containers and for excavation of the underground system of tunnels exists today (Hózer et al. 2010; Kautsky et al. 2013).

**Fig. 3:**
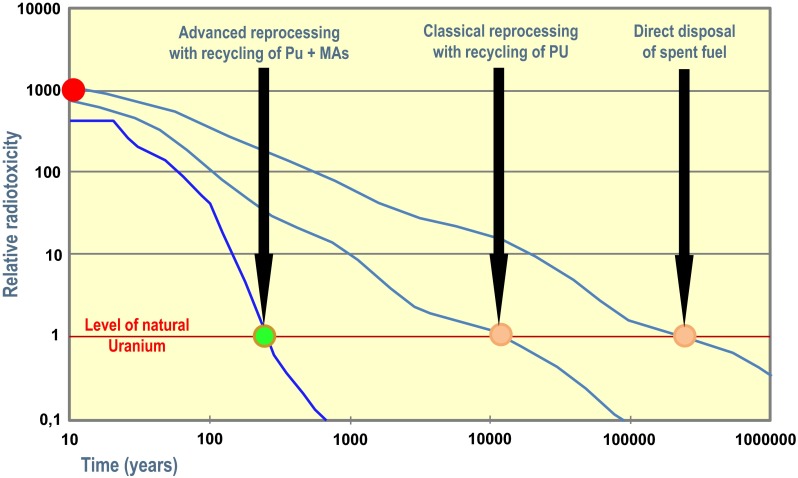
Radiotoxicity of radioactive waste

The European Academies Science Advisory Board recently released the report on “Management of spent nuclear fuel and its waste” (EASAC 2014). The report discusses the challenges associated with different strategies to manage spent nuclear fuel, in respect of both open cycles and steps towards closing the nuclear fuel cycle. It integrates the conclusions on the issues raised on sustainability, safety, non-proliferation and security, economics, public involvement and on the decision-making process. Recently Vandenbosch et al. (2015) critically discussed the issue of confidence in the indefinite storage of nuclear waste. One complication of the nuclear waste storage problem is that the minor actinides represent a high activity (see Fig. 3) and pose non-proliferation issues to be handled safely in a civil used plant. This might be a difficult challenge if the storage is to be operated economically together with the fuel fabrication.

The open (or ‘once through’) cycle only uses part of the energy stored in the fuel, whilst effectively wasting substantial amounts of energy that could be recovered through recycling. The conventional closed fuel cycle strategy uses the reprocessing of the spent fuel following interim storage. The main components which can be further utilised (U and Pu) are recycled to fuel manufacturing (MOX (Mixed Oxide) fuel fabrication), whilst the smaller volume of residual waste in appropriately conditioned form—e.g. vitrified and encapsulated—is disposed of in deep geological repositories.

The advanced closed fuel cycle strategy is similar to the conventional one, but within this strategy the minor actinides are also removed during reprocessing. The separated isotopes are transmuted in combination with power generation and only the net reprocessing wastes and those conditioned wastes generated during transmutation will be, following appropriate encapsulation, disposed of in deep geological repositories. The main factor that determines the overall storage capacity of a long-term repository is the heat content of nuclear waste, not its volume. During the anticipated repository time, the specific heat generated during the decay of the stored HLW must always stay below a dedicated value prescribed by the storage concept and the geological host information. The waste that results from reprocessing spent fuel from thermal reactors has a lower heat content (after a period of cooling) than does the spent fuel itself. Thus, it can be stored more densely.

A modern light water reactor of 1 GWe capacity will typically discharge about 20–25 tonnes of irradiated fuel per year of operation. About 93–94 % of the mass of typical uranium oxide irradiated fuel comprises uranium (mostly ^238^U), with about 4–5 % fission products and ~1 % plutonium. About 0.1–0.2 % of the mass comprises minor actinides (neptunium, americium and curium). These latter elements accumulate in nuclear fuel because of neutron capture, and they contribute significantly to decay heat loading and neutron output, as well as to the overall radiotoxic hazard of spent fuel. Although the total minor actinide mass is relatively small—20 to 25 kg per year from a 1 GWe LWR—it has a disproportionate impact on spent fuel disposal because of its long radioactive decay times (OECD Nuclear Energy Agency 2013).

### Generation IV development

To address the issue of sustainability of nuclear energy, in particular the use of natural resources, fast neutron reactors (FNRs) must be developed, since they can typically multiply by over a factor 50 the energy production from a given amount of uranium fuel compared to current reactors. FNRs, just as today’s fleet, will be primarily dedicated to the generation of fossil-free base-load electricity. In the FNR the fuel conversion ratio (FCR) is optimised. Through hardening the spectrum a fast reactor can be designed to burn minor actinides giving a FCR larger than unity which allows *breeding* of fissile materials. FNRs have been operated in the past (especially the Sodium-cooled Fast Reactor in Europe), but today’s safety, operational and competitiveness standards require the design of a new generation of fast reactors. Important research and development is currently being coordinated at the international level through initiatives such as GIF.

In 2002, six reactor technologies were selected which GIF believe represent the future of nuclear energy. These were selected from the many various approaches being studied on the basis of being clean, safe and cost-effective means of meeting increased energy demands on a sustainable basis. Furthermore, they are considered being resistant to diversion of materials for weapons proliferation and secure from terrorist attacks. The continued research and development will focus on the chosen six reactor approaches. Most of the six systems employ a closed fuel cycle to maximise the resource base and minimise high-level wastes to be sent to a repository. Three of the six are fast neutron reactors (FNR) and one can be built as a fast reactor, one is described as epithermal, and only two operate with slow neutrons like today’s plants. Only one is cooled by light water, two are helium-cooled and the others have lead–bismuth, sodium or fluoride salt coolant. The latter three operate at low pressure, with significant safety advantage. The last has the uranium fuel dissolved in the circulating coolant. Temperatures range from 510 to 1000 °C, compared with less than 330 °C for today’s light water reactors, and this means that four of them can be used for thermochemical hydrogen production.

The sizes range from 150 to 1500 MWe, with the lead-cooled one optionally available as a 50–150 MWe “battery” with long core life (15–20 years without refuelling) as replaceable cassette or entire reactor module. This is designed for distributed generation or desalination. At least four of the systems have significant operating experience already in most respects of their design, which provides a good basis for further research and development and is likely to mean that they can be in commercial operation well before 2030. However, when addressing non-proliferation concerns it is significant that fast neutron reactors are not conventional fast breeders, i.e. they do not have a blanket assembly where plutonium-239 is produced. Instead, plutonium production happens to take place in the core, where burn-up is high and the proportion of plutonium isotopes other than Pu-239 remains high. In addition, new reprocessing technologies will enable the fuel to be recycled without separating the plutonium.

In January 2014, a new GIF Technology Roadmap Update was published.^4^ It confirmed the choice of the six systems and focused on the most relevant developments of them so as to define the research and development goals for the next decade. It suggested that the Generation IV technologies most likely to be deployed first are the SFR, the lead-cooled fast reactor (LFR) and the very high temperature reactor technologies. The molten salt reactor and the GFR were shown as furthest from demonstration phase.

Europe, through sustainable nuclear energy technology platform (SNETP) and ESNII, has defined its own strategy and priorities for FNRs with the goal to demonstrate Generation IV reactor technologies that can close the nuclear fuel cycle, provide long-term waste management solutions and expand the applications of nuclear fission beyond electricity production to hydrogen production, industrial heat and desalination; The SFR as a proven concept, as well as the LFR as a short-medium term alternative and the GFR as a longer-term alternative technology. The French Commissariat à l’Energie Atomique (CEA) has chosen the development of the SFR technology. Astrid (Advanced Sodium Technological Reactor for Industrial Demonstration) is based on about 45 reactor-years of operational experience in France and will be rated 250 to 600 MWe. It is expected to be built at Marcoule from 2017, with the unit being connected to the grid in 2022.

Other countries like Belgium, Italy, Sweden and Romania are focussing their research and development effort on the LFR whereas Hungary, Czech Republic and Slovakia are investing in the research and development on GFR building upon the work initiated in France on GFR as an alternative technology to SFR. Allegro GFR is to be built in eastern Europe, and is more innovative. It is rated at 100 MWt and would lead to a larger industrial demonstration unit called GoFastR. The Czech Republic, Hungary and Slovakia are making a joint proposal to host the project, with French CEA support. Allegro is expected to begin construction in 2018 operate from 2025. The industrial demonstrator would follow it.

In mid-2013, four nuclear research institutes and engineering companies from central Europe’s Visegrád Group of Nations (V4) agreed to establish a centre for joint research, development and innovation in Generation IV nuclear reactors (the Czech Republic, Hungary, Poland and Slovakia) which is focused on gas-cooled fast reactors such as Allegro.

The MYRRHA (Multi-purpose hYbrid Research Reactor for High-tech Applications)^5^ project proposed in Belgium by SCK•CEN could be an Experimental Technological Pilot Plant (ETPP) for the LFR technology. Later, it could become a European fast neutron technology pilot plant for lead and a multi-purpose research reactor. The unit is rated at 100 thermal MW and has started construction at SCK-CEN’s Mol site in 2014 planned to begin operation in 2023. A reduced-power model of Myrrha called Guinevere started up at Mol in March 2010. ESNII also includes an LFR technology demonstrator known as Alfred, also about 100 MWt, seen as a prelude to an industrial demonstration unit of about 600 MWe. Construction on Alfred could begin in 2017 and the unit could start operating in 2025.

Research and development topics to meet the top-level criteria established within the GIF forum in the context of simultaneously matching economics as well as stricter safety criteria set-up by the WENRA FNR demand substantial improvements with respect to the following issues:

- Primary system design simplification,
- Improved materials,
- Innovative heat exchangers and power conversion systems,
- Advanced instrumentation, in-service inspection systems,
- Enhanced safety,

and those for fuel cycle issues pertain to:

- Partitioning and transmutation,
- Innovative fuels (including minor actinide-bearing) and core performance,
- Advanced separation both via aqueous processes supplementing the PUREX process as well as pyroprocessing, which is mandatory for the reprocessing of the high MA-containing fuels,
- Develop a final depository.

Beyond the research and development, the demonstration projects mentioned above are planned in the frame of the SET-Plan ESNII for sustainable fission. In addition, supporting research infrastructures, irradiation facilities, experimental loops and fuel fabrication facilities, will need to be constructed.

Regarding transmutation, the accelerator-driven transmutation systems (ADS) technology must be compared to FNR technology from the point of view of feasibility, transmutation efficiency and cost efficiency. It is the objective of the MYRRHA project to be an experimental demonstrator of ADS technology. From the economical point of view, the ADS industrial solution should be assessed in terms of its contribution to closing the fuel cycle. One point of utmost importance for the ADS is its ability for burning larger amounts of minor actinides (the typical maximum in a critical FNR is about 2 %).

The concept of partitioning and transmutation (P&T) has three main goals: reduce the radiological hazard associated with spent fuel by reducing the inventory of minor actinides, reduce the time interval required to reach the radiotoxicity of natural uranium and reduce the heat load of the HLW packages to be stored in the geological disposal hence reducing the foot print of the geological disposal.

Advanced management of HLW through P&T consists in advanced separation of the minor actinides (americium, curium and neptunium) and some fission products with a long half-life present in the nuclear waste and their transmutation in dedicated burners to reduce the radiological and heat loads on the geological disposal. The time scale needed for the radiotoxicity of the waste to drop to the level of natural uranium will be reduced from a ‘geological’ value (300 000 years) to a value that is comparable to that of human activities (few hundreds of years) (OECD/NEA 2006; OECD 2012; PATEROS 2008^6^). Transmutation of the minor actinides is achieved through fission reactions and therefore fast neutrons are preferred in dedicated burners.

At the European level, four building blocks strategy for Partitioning and Transmutation have been identified. Each block poses a serious challenge in terms of research & development to be done in order to reach industrial scale deployment. These blocks are:

- Demonstration of advanced reprocessing of spent nuclear fuel from LWRs, separating Uranium, Plutonium and Minor Actinides;
- Demonstration of the capability to fabricate at semi-industrial level dedicated transmuter fuel heavily loaded in minor actinides;
- Design and construct one or more dedicated transmuters;
- Fabrication of new transmuter fuel together with demonstration of advanced reprocessing of transmuter fuel.

MYRRHA will support this Roadmap by playing the role of an ADS prototype (at reasonable power level) and as a flexible irradiation facility providing fast neutrons for the qualification of materials and fuel for an industrial transmuter. MYRRHA will be not only capable of irradiating samples of such inert matrix fuels but also of housing fuel pins or even a limited number of fuel assemblies heavily loaded with MAs for irradiation and qualification purposes.

### Options for nuclear fusion beyond 2050

Nuclear fusion research, on the basis of magnetic confinement, considered in this report, has been actively pursued in Europe from the mid-60s. Fusion research has the goal to achieve a clean and sustainable energy source for many generations to come. In parallel with basic high-temperature plasma research, the fusion technology programme is pursued as well as the economy of a future fusion reactor (Ward et al. 2005; Ward 2009; Bradshaw et al. 2011). The goal-oriented fusion research should be driven with an increased effort to be able to give the long searched answer to the open question, “will fusion energy be able to cover a major part of mankind’s electricity demand?”. ITER, the first fusion reactor to be built in France by the seven collaborating partners (Europe, USA, Russia, Japan, Korea, China, India) is hoped to answer most of the open physics and many of the remaining technology/material questions. ITER is expected to start operation of the first plasma around 2020 and D-T operation 2027.

The European fusion research has been successful through the organisation of EURATOM to which most countries in Europe belong (the fission programme is also included in EURATOM). EUROfusion, the European Consortium for the Development of Fusion Energy, manages European fusion research activities on behalf of EURATOM. The organisation of the research has resulted in a well-focused common fusion research programme. The members of the EUROfusion^7^ consortium are 29 national fusion laboratories. EUROfusion funds all fusion research activities in accordance with the “EFDA Fusion electricity. Roadmap to the realisation of fusion energy” (EFDA 2012, Fusion electricity). The Roadmap outlines the most efficient way to realise fusion electricity. It is the result of an analysis of the European Fusion Programme undertaken by all Research Units within EUROfusion’s predecessor agreement, the European Fusion Development Agreement, EFDA.

The most successful confinement concepts are toroidal ones like tokamaks and helical systems like stellarators (Wagner 2012, 2013). To avoid drift losses, two magnetic field components are necessary for confinement and stability—the toroidal and the poloidal field component. Due to their superposition, the magnetic field winds helically around a system of nested toroids. In both cases, tokamak and stellarator, the toroidal field is produced by external coils; the poloidal field arises from a strong toroidal plasma current in tokamaks. In case of helical systems all necessary fields are produced externally by coils which have to be superconductive when steady-state operation is intended. Europe is constructing the most ambitious stellarator, Wendelstein 7-X in Germany. It is a fully optimised system with promising features. W7-X goes into operation in 2015.^8^

**Fig. 4:**
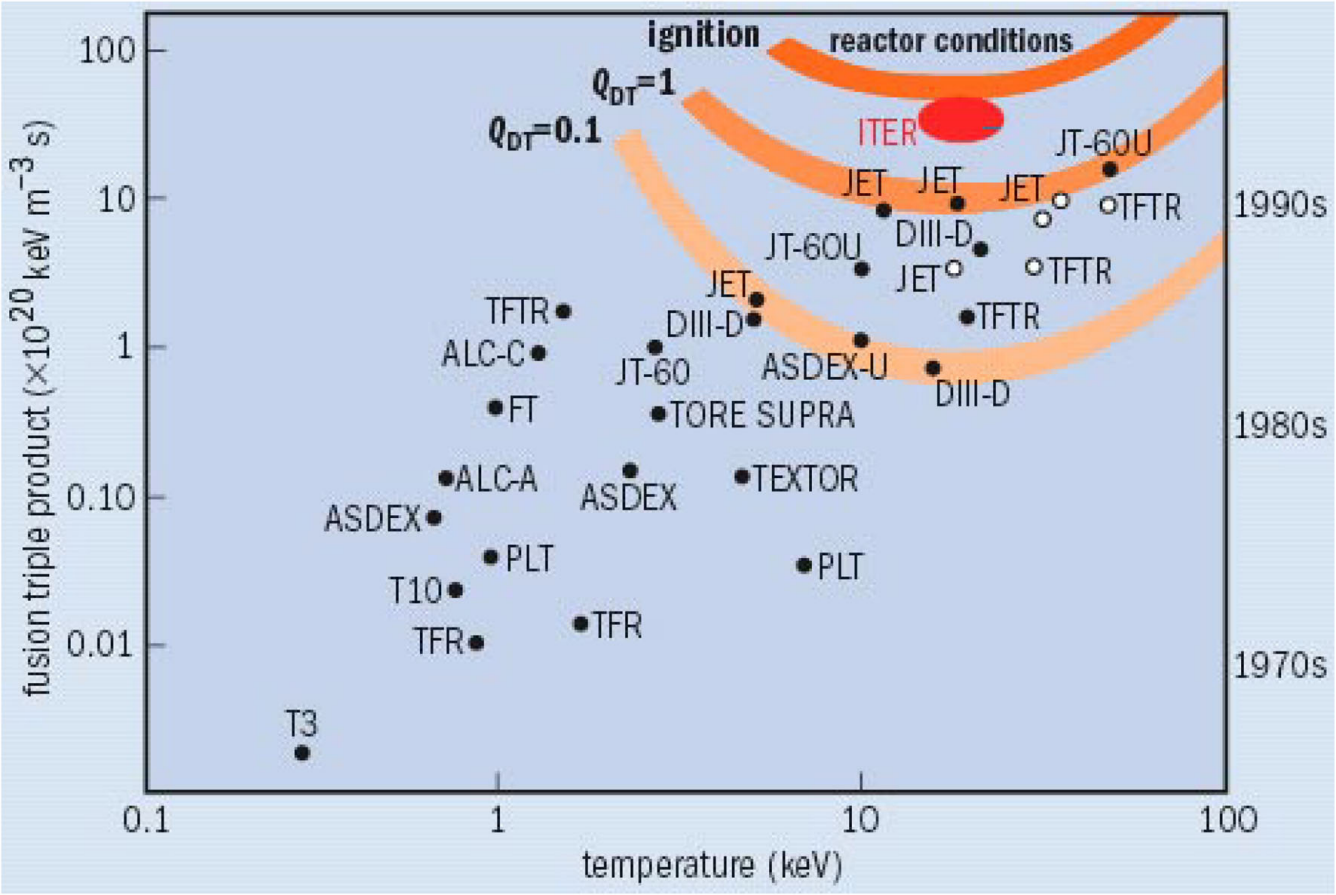
Progress in fusion parameters. Derived in 1955, the Lawson criterion specifies the conditions that must be met for fusion to produce a net energy output (1 keV × 12 million K). From this, a fusion “triple product” can be derived, which is defined as the product of the plasma ion density, ion temperature and energy confinement time. This product must be greater than about 6 × 10^21^ keV m^−3^ s for a deuterium–tritium plasma to ignite. Due to the radioactivity associated with tritium, today’s research tokamaks generally operate with deuterium only (*solid dots*). The large tokamaks JET(EU) and TFTR(US), however, have used a deuterium–tritium mix (*open dots*). The rate of increase in tokamak performance has outstripped that of Moore’s law for the miniaturisation of silicon chips (Pitts et al. 2006). Many international projects (their names are given by acronyms in the figure) have contributed to the development of fusion plasma parameters and the progress in fusion research which serves as the basis for the ITER design

Fusion research has now reached plasma parameters needed for a fusion reactor, even if not all parameters are reached simultaneously in a single plasma discharge (see Fig. 4). Plotted is the triple product n•τ_E•_T_i_ composed of the density n, the confinement time τ_E_ and the ion temperature T_i_. For ignition of a deuterium–tritium plasma, when the internal α-particle heating from the DT-reaction takes over and allows the external heating to be switched off, the triple product has to be about >6 × 10^21^ m^−3^ s keV). The record parameters given as of today are shown together with the fusion experiment of its achievement in Fig. 4. The achieved parameters and the missing factors to the ultimate goal of a fusion reactor are summarised below:

- Temperature: 40 keV achieved (JT-60U, Japan); the goal is surpassed by a factor of two
- Density n surpassed by factor 5 (C-mod,USA; LHD,Japan)
- Energy confinement time: a factor of 4 is missing (JET, Europe)
- Fusion triple product (see Fig. 4: a factor of 6 is missing (JET, Europe)
- The first scientific goal is achieved: Q (fusion power/external heating power) ~1 (0,65) (JET, Europe)
- D-T operation without problems (TFTR (USA), JET, small tritium quantities have been used, however)
- Maximal fusion power for short pulse: 16 MW (JET)
- Divertor development (ASDEX, ASDEX-Upgrade, Germany)
- Design for the first experimental reactor complete (ITER, see below)
- The optimisation of stellarators (W7-AS, W7-X, Germany)

After 50 years of fusion research there is no evidence for a fundamental obstacle in the basic physics. But still many problems have to be overcome as detailed below:

#### Critical issues in fusion plasma physics based on magnetic confinement

- confine a plasma magnetically with 1000 m^3^ volume,
- maintain the plasma stable at 2–4 bar pressure,
- achieve 15 MA current running in a fluid (in case of tokamaks, avoid instabilities leading to disruptions),
- find methods to maintain the plasma current in steady-state,
- tame plasma turbulence to get the necessary confinement time,
- develop an exhaust system (divertor) to control power and particle exhaust, specifically to remove the α-particle heat deposited into the plasma and to control He as the fusion ash.

#### Critical issues in fusion plasma technology

- build a system with 200 MKelvin in the plasma core and 4 Kelvin about 2 m away,
- build magnetic system at 6 Tesla (max field 12 Tesla) with 50 GJ energy,
- develop heating systems to heat the plasma to the fusion temperature and current drive systems to maintain steady-state conditions for the tokamak,
- handle neutron-fluxes of 2 MW/m^2^ leading to 100 dpa in the surrounding material,
- develop low activation materials,
- develop tritium breeding technologies,
- provide high availability of a complex system using an appropriate remote handling system,
- develop the complete physics and engineering basis for system licensing.

#### The goals of ITER

**Fig. 5:**
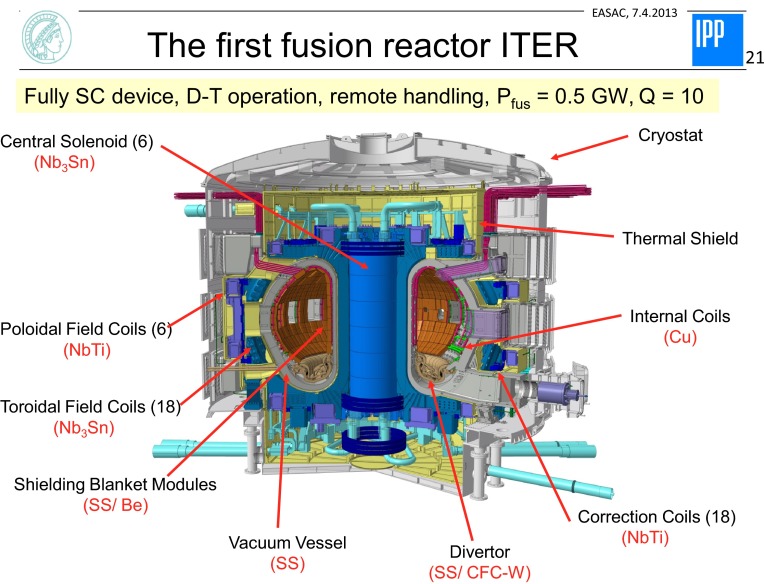
Schematic layout of the ITER reactor experiment (from www.iter.org)

The major goals of ITER (see Fig. 5) in physics are to confine a D-T plasma with α-particle self-heating dominating all other forms of plasma heating, to produce about ~500 MW of fusion power at a gain *Q* = fusion power/external heating power, of about 10, to explore plasma stability in the presence of energetic α-particles, and to demonstrate ash-exhaust and burn control.

In the field of technology, ITER will demonstrate fundamental aspects of fusion as the self-heating of the plasma by alpha-particles, show the essentials to a fusion reactor in an integrated system, give the first test a breeding blanket and assess the technology and its efficiency, breed tritium from lithium utilising the D-T fusion neutron, develop scenarios and materials with low T-inventories. Thus ITER will provide strong indications for vital research and development efforts necessary in the view of a demonstration reactor (DEMO). ITER will be based on conventional steel as structural material. Its inner wall will be covered with beryllium to surround the plasma with low-Z metal with low inventory properties. The divertor will be mostly from tungsten to sustain the high α-particle heat fluxes directed onto target plates situated inside a divertor chamber. An important step in fusion reactor development is the achievement of licensing of the complete system.

**Fig. 6:**
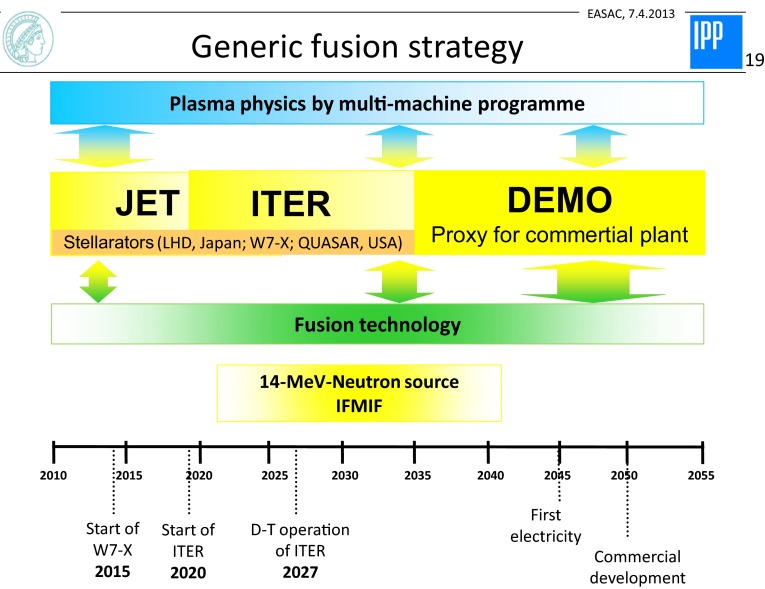
Fusion time strategy towards the fusion reactor on the net (EFDA 2012, Fusion electricity. A roadmap to the realisation of fusion energy)

The rewards from fusion research and the realisation of a fusion reactor can be described in the following points:

- fusion has a tremendous potential thanks to the availability of deuterium and lithium as primary fuels. But as a recommendation, the fusion development has to be accelerated,
- there is a clear roadmap to commercialise fusion as shown by Fig. 6 (EFDA 2012). The major lines are from the presently largest tokamak JET via ITER (a tokamak) to the demonstration reactor DEMO. This line is accompanied by the multi-machine science programme including concept improvement via the family of helical systems.

In addition, there is the fusion technology programme and its material branch, which ultimately need a neutron source to study the interaction with 14 MeV neutrons. For this purpose, a spallation source IFMIF is presently under design. As a recommendation, ways have to be found to accelerate the fusion development. In general, with ITER, IFMIF and the DEMO, the programme will move away from plasma science more towards technology orientation. After the ITER physics and technology programme—if successful—fusion can be placed into national energy supply strategies. With fusion, future generations can have access to a clean, safe and (at least expected of today) economic power source.

## Summary

The fission nuclear power continues to be an essential part of the low-carbon electricity generation in the world for decades to come. There are breakthrough possibilities in the development of new generation nuclear reactors where the life-time of the nuclear waste can be reduced to some hundreds of years instead of the present time-scales of hundred thousand of years. Research on the fourth generation reactors is needed for the realisation of this development. For the fast nuclear reactors a substantial research and development effort is required in many fields—from material sciences to safety demonstration—to attain the envisaged goals. Fusion provides a long-term vision for an efficient energy production. The fusion option for a nuclear reactor for efficient production of electricity should be vigorously pursued on the international arena as well as within the European energy roadmap to reach a decision point which allows to critically assess this energy option.

## Box 1 Explanations of abbreviations used in this article

ADS: Accelerator-driven transmutation systems
AGR: Advanced gas-cooled reactor
ASTRID: Advanced sodium technological reactor for industrial demonstration
CEA: Commissariat l´Energie Atomique
DEMO: Demonstration power plant
ESNII2000: European sustainable nuclear industrial initiative for sustainable fission
ETTP: Experimental technological pilot plant
EURATOM: The European Atomic Energy Community
IAEA: International Atomic Energy Agency
FNR: Fast neutron reactor
GFR: Gas-cooled fast reactor
GIF: Generation IV international forum
GWe: Giga watt energy
HLW: High-level radioactive waste
IFMIF: International fusion materials irradiation facility
INPRO: International project on innovative nuclear reactors and fuel cycles
ITER: International thermonuclear experimental reactor or from latin “the way”
LFR: Lead-cooled fast reactor
LWR: Light water reactor
MOX: Mixed oxide fuel
MYRRHA: Multi-purpose hybrid research reactor for high-tech applications
P&T: Partitioning and transmutation
PATEROS: Partitioning and transmutation European roadmap for sustainable nuclear energy
PUREX process: Plutonium and Uranium extraction process
*Q*-value: Fusion energy gain factor (*P*_fus_/*P*_heat_)
SET-plan: Strategic energy technology plan
SFR: Sodium-cooled fast reactor
SNETP: Sustainable nuclear energy technology platform

## Footnotes

1 http://www.iea.org/.

2 https://cnpp.iaea.org/pages/index.htm.

3 GenIV International forum: (http://www.gen-4.org/index.html).

4 https://www.gen-4.org/gif/jcms/c_60729/technology-roadmap-update-2013.

5 http://myrrha.sckcen.be/.

6 www.sckcen.be/pateros/.

7 https://www.euro-fusion.org/.

8 https://www.ipp.mpg.de/ippcms/de/pr/forschung/w7x/index.html.
